# Why just fly?

**DOI:** 10.1080/19336934.2025.2593725

**Published:** 2025-11-24

**Authors:** Peter K. Dearden

**Affiliations:** Genomics Aotearoa and Biochemistry Department, University of Otago, Dunedin, Aotearoa-New Zealand

**Keywords:** *Drosophila*, insects, food production, insect apocalypse, evolution

## Abstract

*Drosophila melanogaster* is an incredible model system, providing tools and technologies that allow careful, effective, and reproducible research. This experimental approach, and the genetic tools and techniques available in *Drosophila* are desperately needed for the study of other insects, a hugely diverse group of huge importance to natural and productive ecosystems. For those of you with the skills and ‘*Drosophila* mindset’, studying other insects may help us understand diversity, improve the security of food production, and help avoid the current, worrying, insect apocalypse.

## Why flies are awesome

I started my research career in flies, and I still regard them as the best place to answer many biological questions. A great deal of what we know about genes, what they do, and how they work comes from flies. Biomedical science would be lost without the knowledge developed and still being produced from *Drosophila melanogaster* [1–4].

The ethos behind the *Drosophila* community has always been a key driver of its success [5,6]. The sharing of fly lines and reagents, and the open sharing of results and progress at conferences, has made the field an exemplar of how to do good science. This is evident in recent studies of reproducibility of *Drosophila* studies [7,8], which have shown a scientific field that is reproducible, self-correcting and driven by data rather than hype. Much of this is driven by extremely stringent reviewing of papers and grants. To my mind, the genetic approach to *Drosophila*, including all the remarkable and fine-scale tools that have been developed to test gene function (for review see [9]), has led to a field that asks good questions and answers them effectively. This attention to detail and high burden of proof make for better publications and better science.

## Bringing the fly!

Given how the *Drosophila* community continues to drive excellent and high-quality science, why should people stop doing it? Most *Drosophila* researchers have traditionally focused on biomedical science. Much of what has been achieved is applicable broadly in biology, but the driving focus has been improving the human condition- especially through medical technologies. Now, clearly, there is nothing wrong with that, but I think we have neglected the importance of insects in our ecosystems, food production systems, and in life’s diversity.

Let’s, for a moment, consider honey bees (*Apis mellifera*). Honeybees underpin about US$9 billion in hive products worldwide, and the annual worldwide value of their pollination efforts is estimated between US$235 and US$285 billion [10]. They are challenged by pests and diseases￼, which in the USA have led to the loss of up to 40% of colonies each year [11].

Given the importance of this one species of insect, why do we not have all the tools and technologies that are available in *Drosophila*? While RNAi [12], and CRISPR/cas ([13,14]) editing are available, we do not yet have over-expression systems or the ability to map the neural pathways of the brain [15,16]. With the remarkable social biology of these insects and their importance to us as a species, can you imagine the insights that could be drawn if we did have those tools and abilities? Too much of the science of honey bees is driven by gene expression studies that are not followed up with manipulative experiments because the tools are not available, or are too hard. Yes, honey bees are a bit harder to grow than *Drosophila*, and yes, they sting, but we are never going to be able to understand them properly, improve their health, and deploy them effectively and sustainably without the rigorous science used in *Drosophila*.

**Figure 1.**
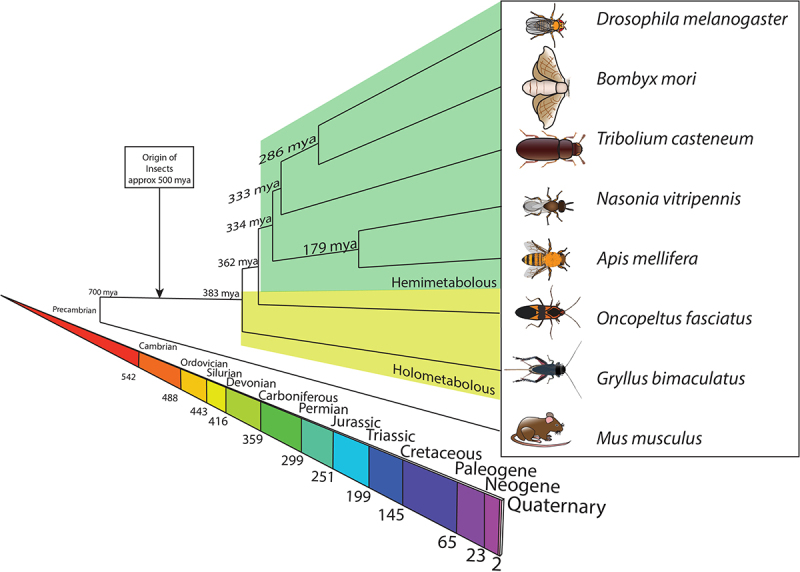
Geological time frame indicating the dates at which the separation of groups of insects and other animals diverged (dates from [18]), as well as some of the key systems used for studies of insect biology.

I am not trying to imply that transferring the tools and approaches from *Drosophila* to honeybees is easy; the tools that have been developed have been hard-won, and the genetic and social structures of honeybees are confounding. We do, however, think nothing of suggesting that work on flies is relevant to human health, a distance of probably 700 million years [17,18]; surely the 344 million gap [19] to honeybees is also bridgeable? (Figure 1)

## Diversity

Beetles, Coleoptera, are supposed to be the most speciose group of animals on earth. We suspect that we have identified only 20% of them, and there may be as many as 5.5 million species [20]. J.B.S. Haldane’s joke, that the creator has an ‘inordinate fondness for beetles’, also applies to flies (Diptera) and bees, ants and wasps (Hymenoptera). Indeed, current estimates suggest that Coleoptera and Hymenoptera are in a neck-and-neck race for the largest number of species [21].

**Figure 2.**
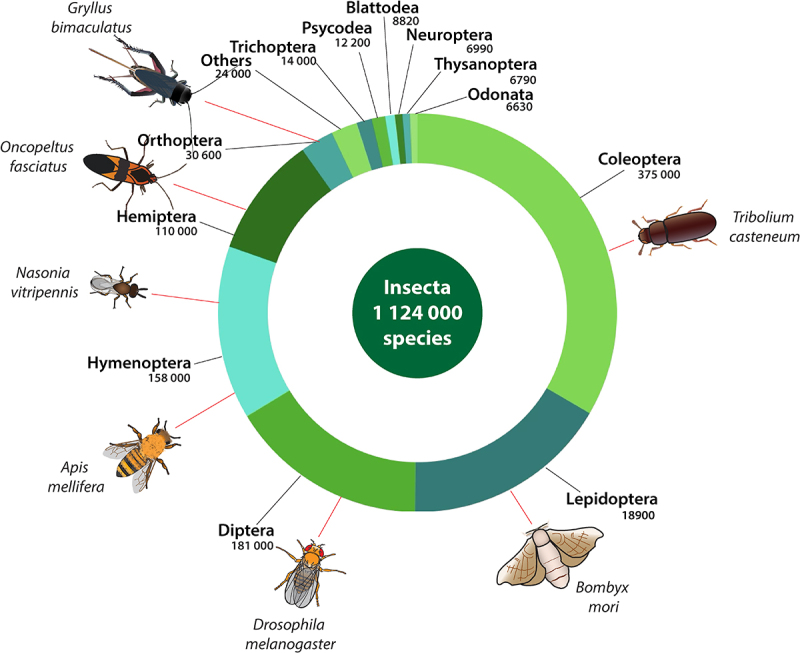
The diversity of insects. Insect orders and their number of described species, as well as the placement of key species for insect biology studies.

Diptera, Coleoptera and Hymenoptera, along with the Lepidoptera and some others [22], make up most of the holometabolous insects, but there are also hemimetabolous insects, including aphids, crickets, termites, cockroaches, lice and more (Figure 2). All these groups live in almost all non-marine environments, though there are even some pond skaters that live on the surface of the ocean [23] and seal lice that cope with a mainly marine existence [24]. The adaptation of all these species to a huge range of niches and life history strategies makes for a happy hunting ground for scientists who wish to understand how diversity arises.

Studies of segmentation [25–30], wing patterning [31–33], body colouration [34–36], and limb development [37–39], all in diverse insect species that differ from *Drosophila melanogaster*, form the basis of our understanding of the evolution of shape and form. Diversity, however, goes beyond that. In the Hymenoptera alone we have the repeated evolution of complex social structures [40], the evolution of multiple varieties of parasitism [41], the evolution of smaller body size to the point where the neurons of the brain jettison the cell nucleus to reduce cell size [42,43], and species that clones males of a separate species to maintain its worker caste [44].

Understanding the remarkable diversity of insect biology requires the rigour and experimental approaches of *Drosophila* science. Some of these ‘insect quirks’ will rock our understanding of the basic biology we think all animals share. Imagine what we could learn if we could, gene by gene, dissect these traits, as we would in *Drosophila*?

## Feeding the world

The miracle of biomedical science, much underpinned by work in *Drosophila*, has extended the life span and reproductive success of humans, unequally, around the world. As a result, the planet is filled with billions of humans, with billions more on the way. The human population of Earth is projected to reach 9.1 billion in 2050. If our food production systems do not keep up with population growth, then the biggest issue for human health will be starvation. Finding new ways to grow food that are less damaging, more efficient and resilient in the face of climate change will be crucial in the next few years. Insects, of course, are a big part of this.

Insect pests damage crops and consume and spoil stored food. Losses due to insect damage are estimated at US$220 billion annually (International Plant Protection Convention), with a mean of 42.1% of agricultural crops destroyed each year by insect pests [45]. Insects are also clearly required for food production, such as pollination, and ecosystem services, such as pest control, that we do not even measure. Our current response to all sorts of insect-related issues in agriculture appears to be the application of long-lasting broad-range insecticides [46–48], which are damaging to pest and beneficial insects alike. While change is happening, with extensive use of Bt crops (reviewed in [49]), and more specific insecticides (for example see [50,51]) and RNAi approaches (for example see [52,53]) on the way, we desperately need more tools to manage pest and beneficial insects.

More specific technologies for pests are needed. Gene drives, for example, have been developed in a few dipteran species to control insects that spread human disease [54–57], or in species, such as *Drosophila suzukii* [58,59], that are crop pests but close to *D. melanogaster*. Such technologies may be helpful in crop pests [60,61], or invasive species [62], if they can be shown to be safe and effective. More specific toxins for use as selective insecticides [63–65] are only going to come about through a deep understanding of the pests involved and the species-specific biology that could provide a target. Biocontrol [66–68] is another option, but identifying bacterial or fungal pathogens, or parasites and predators, is challenging. Again, more knowledge is required, and given the stakes, that knowledge needs to be deep and mechanistic. Work on pest and beneficial insects is, of course, going ahead, but we need more of it, as there are plenty of insects we need to understand, and we need better tools, technologies and ideas.

Insects may also be a good way to produce food – many species have been consumed by humans for centuries [69]. Crickets, or ‘lawn prawns’, are potentially a good source of protein [70], and many insect species can be grown cheaply on agricultural waste, a source of nutrients that we may not be able to ignore. This is a nascent industry that the tools of *Drosophila* genetics may help with – can we make insects with better characteristics for food production? Can we make them more tasty and less spiky? Is *Drosophila* science the key to unlocking a new, and much-needed, food production system?

## The insect apocalypse

In the past few years, ecologists have been warning us of the global loss of insects. Studies such as [71,72] and [73] have shown appalling and unsustainable losses of insect numbers and species. In much of the world, we do not even measure insect numbers, and so have no idea how insects are tracking. What is responsible for this collapse is also not entirely clear, though climate change [74,75], D. L [76]. and the widespread use of broad-range insecticides [77–80] are leading suspects. It seems that we are putting the planet through a mass extinction of insects, sometimes called the Insect apocalypse [81–83].

Insects underpin much of the terrestrial ecosystems of life on Earth [84,85]. They perform much-needed ecosystem services [86] such as clearing waste, pest control, pollination and more. They also form the base of many food webs; the consequences of losing them are predicted in Rachel Carson’s book ‘Silent Spring’ [87]. The impact on our natural and productive landscapes of the decline of insects is not clear, but it is likely devastating.

So we need science to support insects and to stop this decline. Much of this will be ecological, but there is a need for rigorous genetics and molecular biology. Understanding the molecular roots of the insect decline, and finding new ways to manage ecosystems needs the skills of *Drosophila* scientists.

## Come join us!

There is a huge range of questions that insects other than *Drosophila* can help answer. There are even some half-decent experimental systems that have been developed, where some of the *Drosophila* tools and technologies are becoming available (Figure 2). These include red flour beetles (*Tribolium casteneum*) [88–91], Jewel wasps (*Nasonia vitripennis*) [92,93], [94–96], Honey bees (*Apis mellifera*) [12–14,97,98], Milkweed bugs (*Oncopeltus fasciatus*) [27,99–103], Crickets of various sorts [104–110] and even flies close to *Drosophila*, such as *D. suzukii* [111–113], F [114–116]. Perhaps the best example is in mosquitos, where the need to develop new tools to control human disease has led to a raft of transgenic technologies (for examples see [117–119]). Rather than give an exhaustive list of all the species that are being worked on, it’s probably better to say that RNA interference (pioneered in non-model insects by Thom Kaufman [102]) and CRISPR technologies have become key in opening up a range of species to genetic technologies. Technologies for genetic modification that work across species are being developed [120–126], and the genomic resources required for modern genetics are available in many species.

So if you have a desire to use the skills you have as a *Drosophila* geneticist, to do something both challenging and of worldwide importance, consider switching to work on the insects that underpin some of the key problems of our planet, and much of its diversity. And while you think about that, have you met the rest of the Arthropods? They are pretty cool … .

## Data Availability

No new data was generated in this work.
